# Case report: Urgent surgical management of pediatric clear cell sarcoma of the kidney with atrial obstruction

**DOI:** 10.3389/fped.2022.965541

**Published:** 2022-08-17

**Authors:** Alexandra Varga, Gábor Bogáts, Katalin Rácz, Tamás Kovács

**Affiliations:** ^1^Division of Pediatric Surgery, Department of Pediatrics, Albert Szent-Györgyi Clinical Center, University of Szeged, Szeged, Hungary; ^2^Unit of Cardiac Surgery, Department of Internal Medicine, Albert Szent-Györgyi Clinical Center, University of Szeged, Szeged, Hungary; ^3^Department of Pediatrics, Albert Szent-Györgyi Clinical Center, University of Szeged, Szeged, Hungary

**Keywords:** clear cell sarcoma of the kidney, cavoatrial tumor thrombus, atrial obstruction, pediatric oncology, urgent surgery

## Abstract

Clear cell sarcoma of the kidney (CCSK) is an uncommon renal neoplasm of childhood. Progression of intracaval or cavoatrial thrombosis is extremely rare and mostly asymptomatic, treated with neoadjuvant therapy followed by surgery. However, in an unstable patient, acute radical surgical intervention is the treatment of choice. We present a 2-year-old girl diagnosed as having a large left kidney tumor and acute cardiac decompensation *via* cavoatrial thrombotic progression. Urgent radical nephrectomy and removal of tumor thrombus were performed using atriotomy and inferior vena cava (IVC) endarterectomy under cardiopulmonary bypass. Histopathology revealed CCSK. The patient is tumor-free at 9-year follow-up.

## Introduction

Clear cell sarcoma of the kidney (CCSK) is an uncommon renal tumor (RT), comprising 2%−5% of all primary renal malignancies in children (1). Typically, onset occurs between 2 and 4 years of age with a slight male predominance (1). The most common symptoms are abdominal mass, abdominal pain, and hematuria (2). Preoperative diagnosis is challenging, infrequent, and often misdiagnosed until formal pathologic evaluation because of the lack of specific radiologic morphology (3). Histologically, it has a wide diversity of morphologic patterns: myxoid, sclerosing, cellular, epithelioid, palisading, spindle cells, storiform, and anaplastic (2). These forms can mimic other pediatric RTs, resulting in inappropriate or delayed treatment (2). Recently, immunohistochemistry for BCL-6 coreceptor (BCOR) has been shown to be a sensitive and specific marker to distinguish CCSK from its mimics (1, 2).

Clear cell sarcoma of the kidney has a propensity for aggressive behavior and late relapses (2). The most frequent metastatic sites are regional lymph nodes, bone, lung, and liver (2). Cavoatrial thrombotic progression is extremely rare, with only a few pediatric cases reported (4–6). In these cardiologically asymptomatic cases, treatment strategies were based on neoadjuvant chemotherapy followed by surgical resection of the tumor and thrombus (4–6). In contrast, cavoatrial tumor extension may cause acute heart failure, which requires urgent surgical intervention (7). Until now, there has been limited information in the literature concerning this condition. In this case report, we describe the successful management of CCSK with cardiac progression, resulting in acute cardiac decompensation, using cardiopulmonary bypass and deep hypothermic circulatory arrest.

## Case description

A previously healthy 2-year-old girl was urgently admitted to a county hospital with a 3-week history of intermittent abdominal pain, paleness, and general fatigue. Further, on physical examination, a huge left abdominal mass and hepatomegaly were palpable. Electrocardiogram (ECG) showed a right ventricular strain pattern, and laboratory tests revealed anemia and hematuria.

**Figure 1:**
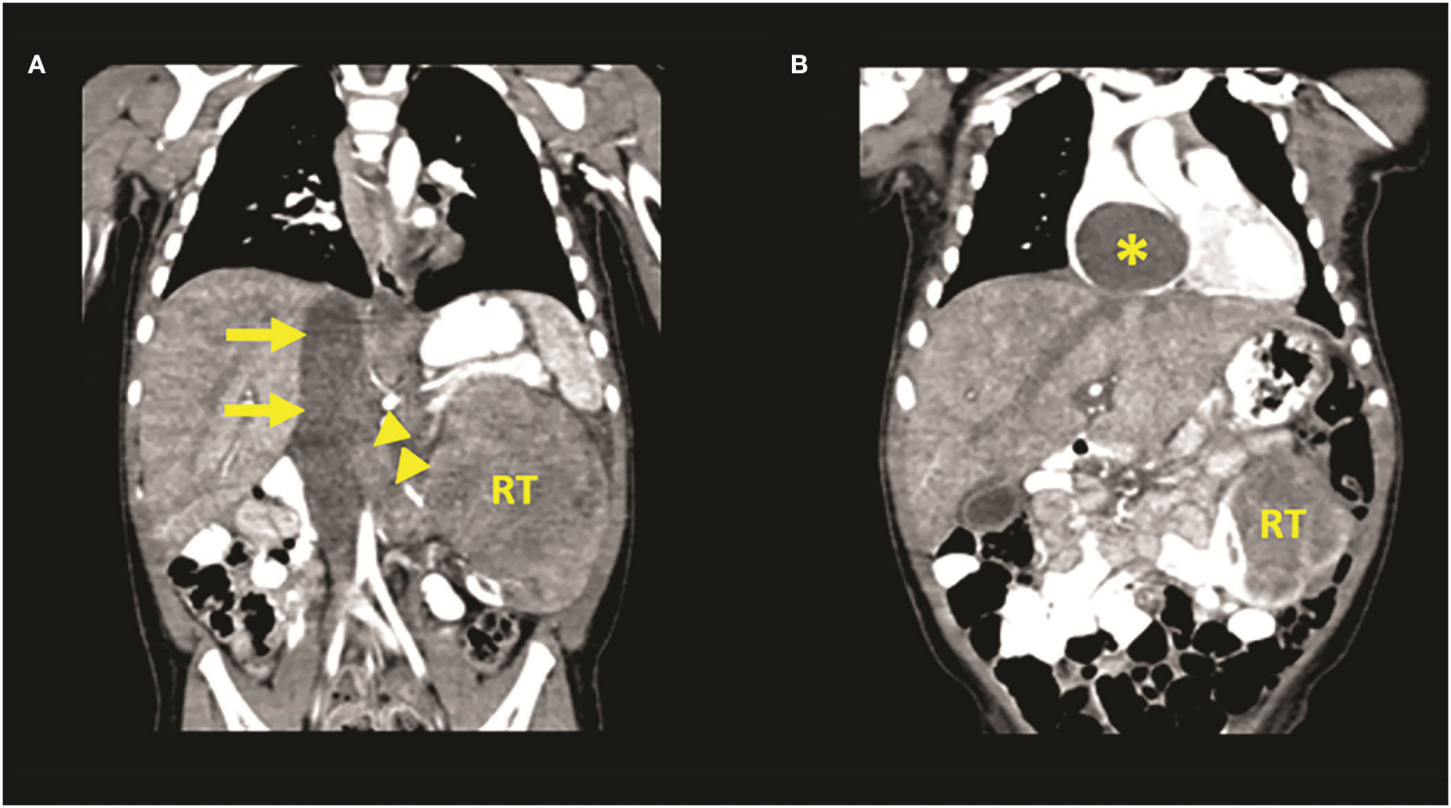
Venous phase of computed tomography (CT) angiography images **(A,B)** showing a left renal tumor (RT) with renal vein (arrowheads) and inferior cava vein tumor thrombus (arrows) extending into the right atrium (asterisk).

Ultrasonography and computed tomography (CT) revealed a large left heterogeneous RT measuring 80 mm × 95 mm × 67 mm, with a tumor thrombus in the inferior vena cava (IVC) extending into the right atrium (Figure 1). No pulmonary or liver metastases were observed. Two-dimensional echocardiography showed an IVC thrombus with blood flow only in a 1–2-mm-wide zone between the thrombus and the vessel wall and a large mass filling the right atrium. The girl was transported to a tertiary pediatric surgical and cardiac surgery center for further treatment. In addition to the abdominal findings, physical examination highlights a worsening of the general condition with mild tachypnea, dyspnea, and tachycardia. Hematuria without coagulation disorders was found. The results of cardiological examination are similar to the results of echocardiogram. Due to its rapid progression, a multidisciplinary team decided on an emergency surgical intervention. Neoadjuvant chemotherapy was not feasible. During preoperative preparation, signs of cardiac decompensation (tachycardia, tachypnea-dyspnea, and somnolence) emerged due to the obstruction of the inflow and outflow of the right atrium, increasing the anesthetic risk of the operation.

**Figure 2:**
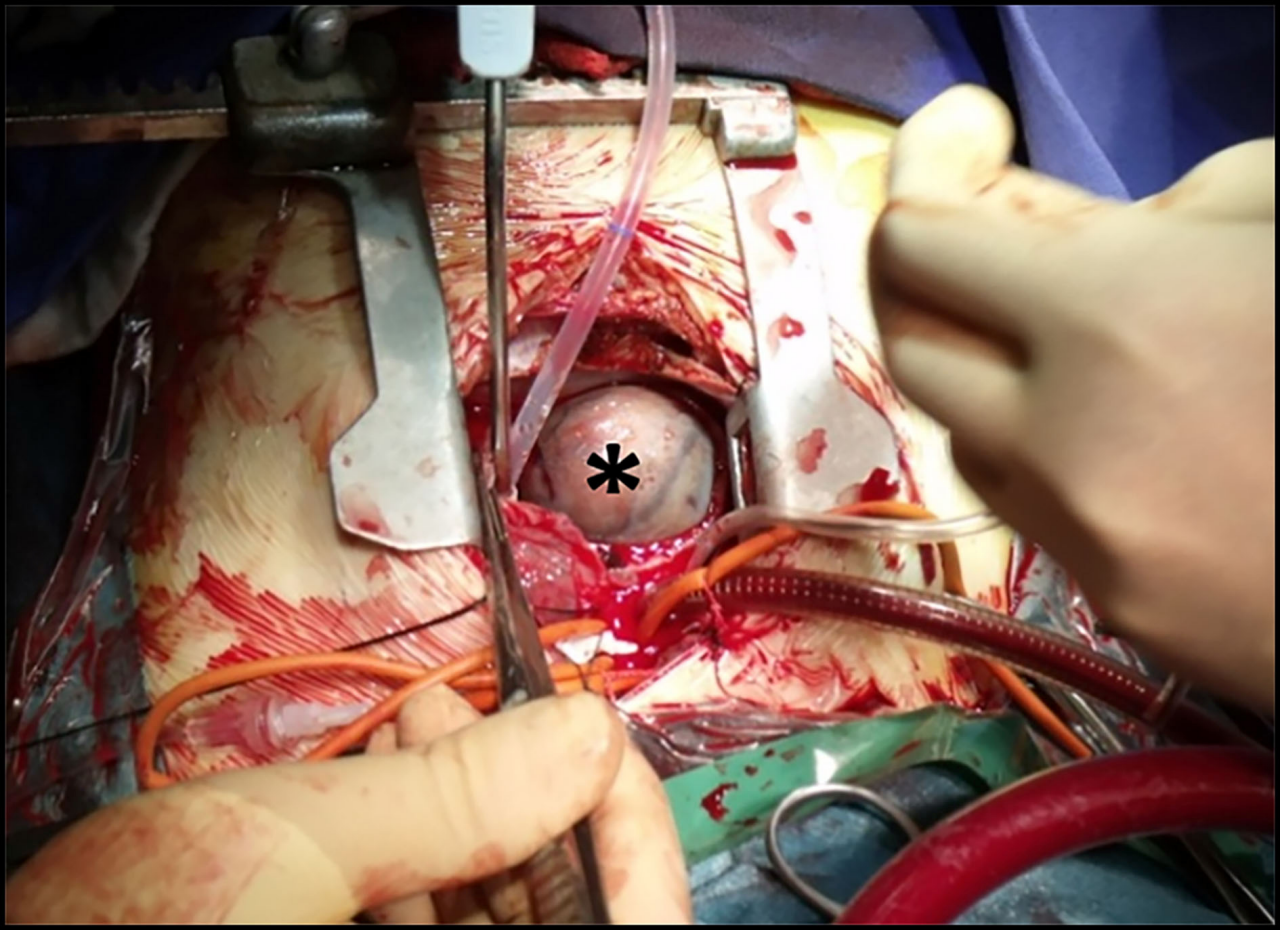
Intraoperative photograph presenting the right atrial tumor thrombus (asterisk) after performing thoracotomy and atriotomy.

The operation was performed simultaneously by the cardiac and pediatric surgical team. After median sternotomy, deep hypothermic cardiopulmonary bypass was initiated with total circulatory arrest. Subsequently, the left radical nephrectomy was performed through the left transverse laparotomy. The renal vein was opened and the tumor thrombus was completely removed, then the renal vein was ligated at the level of the IVC. The next steps were right atriotomy and removal of the tumor thrombus from the right atrium and later from the IVC (Figure 2). As the thrombus was not -adhered to the wall of IVC, complete removal of the tumor was achieved without requiring cavotomy using the endarterectomy technique. In the postoperative period, a retroperitoneal hematoma developed in the heparinized patient and was evacuated. Otherwise, the postoperative course was uneventful. Histopathology confirmed CCSK with cavoatrial tumor thrombus.

**Figure 3:**
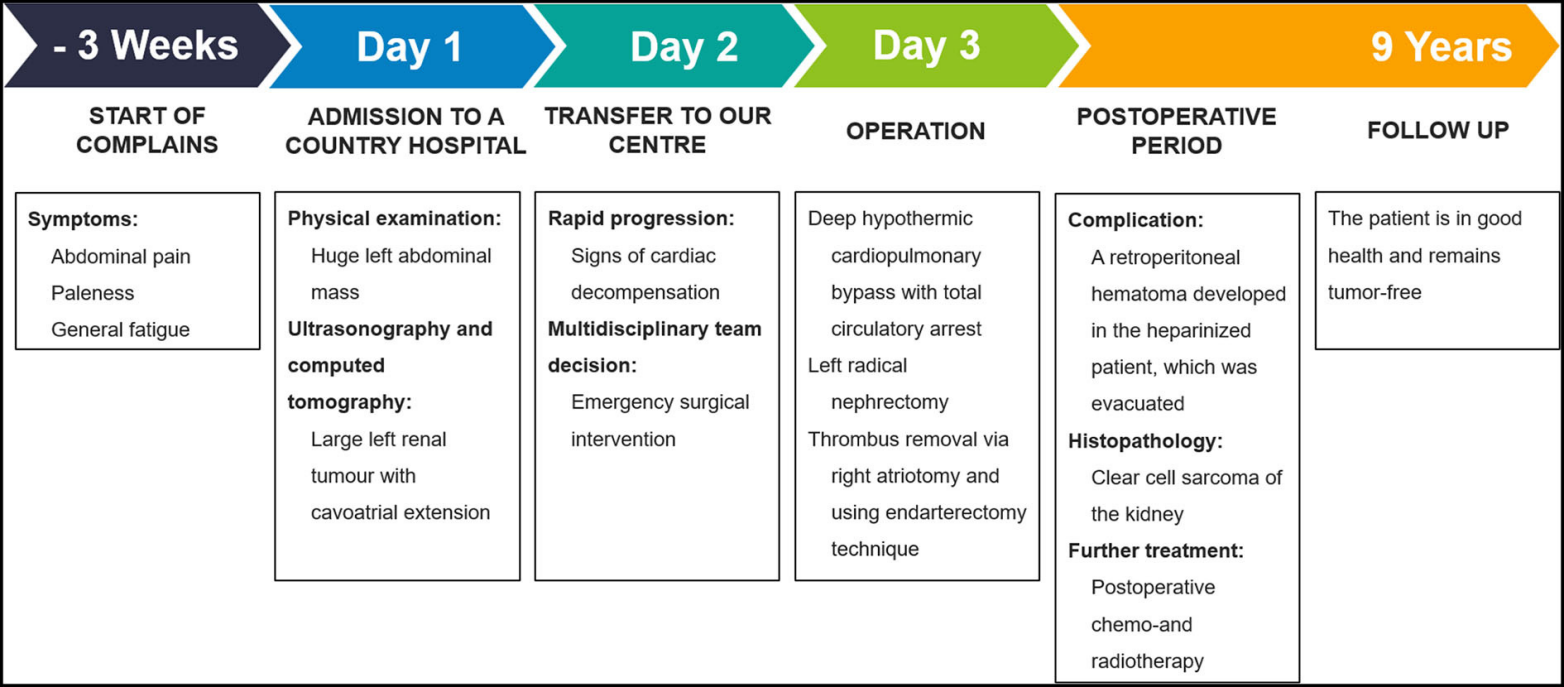
Timeline summarizing the major events of the case.

The patient has received postoperative chemotherapy and radiotherapy, and after 9 years of follow-up, she is in good health and remains tumor-free. Timeline (Figure 3) demonstrates the key events of the case.

## Discussion

Intravascular tumor extension is mainly characteristic of Wilms' tumor (WT) in children (6, 8). In contrast to WT, where intracaval tumor extension occurs in 4%−8% and right atrial progression in 1%−3% of cases, intravascular extension of CCSK is almost unknown and tumor progression into the right atrium is even rarer: three pediatric cases of CCSK with cavoatrial extension have been previously reported in the English literature (4–6, 9).

Most patients with thrombotic progression are asymptomatic, and the diagnosis is only made by imaging studies (7). The presence of a tumor thrombus may be suspected when the patient presents with varicocele, genital edema, abdominal wall collateral vessels, ascites, macroscopic hematuria, renal exclusion, or cardiac murmur (10). Cardiac failure is a rare complication and can be caused by tricuspid stenosis or insufficiency, right ventricular outflow tract obstruction and somnolence, and tachycardia, tachypnea-dyspnea, hypotension, or pleural effusion may be present (11–13). Acute decompensated heart failure is present only among a few cases of patients with WT as early symptoms requiring primary surgical intervention (14). Interestingly, none of the published pediatric CCSK cases with cavoatrial involvement were complicated with this condition (4–6).

Generally, the UMBRELLA SIOP–RTSG 2016 protocol recommends treating pediatric patients with CCSK with neoadjuvant therapy: vincristine and actinomycin for localized disease, supplement with doxorubicin for metastatic disease (1). However, for intravascular thrombotic progression, it has no specific recommendations (1). Surgical guidelines for CCSK proper to WT are recommended (15). Irrespective of disease stage, patients will be treated postoperatively with alternating ifosfamide and cyclophosphamide in combination with etoposide, carboplatin, and doxorubicin (1). Patients with local stages II–III of CCSK are recommended to receive postoperative abdominal radiotherapy (1). For metastatic cases (stage IV), metastasectomy is advised whenever surgical treatment can be performed without mutilation or loss of vital organs (15). Otherwise, radiotherapy is strongly suggested (15).

The prognosis of CCSK was originally considered poor and assigned to the “unfavorable histology” category, however, with current intensive treatment including multiagent chemotherapy and radiation therapy, it has improved the 5-year overall survival rate to 73%−86% (2). Nevertheless, the 4-year survival of stage IV remains poor at 45% (16).

In the published pediatric CCSK cases with cavoatrial involvement treatment strategies were based on WT guidelines involving neoadjuvant therapy followed by delayed *en bloc* resection: primary surgery is indicated in an unstable patient, mostly to avoid a potentially fatal tumor embolus (4–7, 9, 17).

Operative techniques for tumor thrombus elimination in childhood RTs are similar to techniques used in adults with cardiac tumor extension (4). Methods include tumor balloon thrombectomy, endarterectomy, excision *via* atriotomy, cavotomy, IVC resection with or without graft replacement, and resection under vascular isolation or cardiopulmonary bypass with or without cardiac arrest (4–6). Urgent interventions due to acute cardiac failure are infrequent in pediatric RTs with atrial extension: Cristofani et al. (14) presented two patients with WT who needed emergency surgical intervention with this condition.

Due to the poor response, the effect of neoadjuvant therapy in CCSK is questionable as well as it may lead to increased adherence of the tumor to the vessels, which hampers complete resection of the lesions (5, 6). Hence, an early attempt at an *en bloc* tumor resection *via* laparotomy and limited venotomy may be beneficial (6). Our intraoperative findings support early treatment: in the absence of preoperative chemotherapy, the tumor thrombus was firm and not adherent to the vascular wall; therefore, complete resection of the intracaval part was feasible through the right atrium using endarterectomy technique. Cavotomy and its reconstruction could thus be avoided.

The major limitation of this report is the description of a single case. Despite the lack of literature and recommendations in this condition, we strongly believe that our experience gained from the successful treatment of this rare complication can help in similar situations. Although this is a case of an advanced CCSK, the long-term tumor-free status can fill us with confidence that we can guarantee a complete recovery for the child.

This uncommon case reveals that CCSK cases with cavoatrial involvement may require urgent intervention. To the best of our knowledge, this is the first published case in the English literature of a successfully treated intraatrially progressing CCSK, which has caused acute heart failure. In this situation, a multidisciplinary team approach and radical *en bloc* resection are of utmost importance. Furthermore, the lack of neoadjuvant chemotherapy may actually be technically beneficial during surgery.

## Data availability statement

The original contributions presented in the study are included in the article/supplementary material, further inquiries can be directed to the corresponding author.

## Ethics statement

Written informed consent was obtained from the minor(s)' legal guardian/next of kin for the publication of any potentially identifiable images or data included in this article.

## Author contributions

TK and AV contributed to the conception and design of the study. TK, GB, and KR participated in patient care: KR performed the cardiovascular examination, GB performed cardiac surgery, and TK performed the pediatric surgical management of the patient. AV wrote the original draft of this manuscript. TK supervised and revisited this work. All authors have made a substantial, direct, intellectual contribution to the work, and approved it for publication.

## Conflict of interest

The authors declare that the research was conducted in the absence of any commercial or financial relationships that could be construed as a potential conflict of interest.

## Publisher's note

All claims expressed in this article are solely those of the authors and do not necessarily represent those of their affiliated organizations, or those of the publisher, the editors and the reviewers. Any product that may be evaluated in this article, or claim that may be made by its manufacturer, is not guaranteed or endorsed by the publisher.
